# Fatal Gastrointestinal Mucormycosis in a Premature Infant Associated with a Contaminated Dietary Supplement — Connecticut, 2014

**Published:** 2015-02-20

**Authors:** Snigdha Vallabhaneni, Tiffany A. Walker, Shawn R. Lockhart, Dianna Ng, Infectious Diseases Pathology Branch, Tom Chiller, Richard Melchreit, Mary E. Brandt, Rachel M. Smith

**Affiliations:** 1 Division of Foodborne, Waterborne, and Environmental Diseases, National Center for Emerging and Zoonotic Infectious Diseases, CDC; 2 Division of High-Consequence Pathogens and Pathology, National Center for Emerging and Zoonotic Infectious Diseases, CDC; 3 Connecticut Department of Public Health, Hartford, Connecticut

In October 2014, a hospital in Connecticut notified CDC and the Connecticut Department of Public Health of a fatal case of gastrointestinal mucormycosis in a preterm infant. The infant, born at 29 weeks’ gestation and weighing 1,400 grams (about 3 pounds), had developed signs and symptoms initially consistent with necrotizing enterocolitis approximately 1 week after birth. Exploratory laparotomy revealed complete ischemia of the gastrointestinal tract from the esophagus to the rectum; a portion of necrotic cecum was sent for microscopic examination. Following surgery, the infant developed multiple areas of vascular occlusion, including a large clot in the aorta, findings not usually associated with necrotizing enterocolitis. The infant died soon after. Histopathology results from the resected cecum revealed an angioinvasive fungal infection consistent with mucormycosis. Gastrointestinal mucormycosis is an extremely rare fungal infection caused by mold in the order Mucorales. It occurs predominantly in low birth weight infants, patients with diarrhea and malnutrition, and those receiving peritoneal dialysis; mortality is 85% (1). Local investigation revealed that the infant had received a dietary supplement, ABC Dophilus Powder, for 7 days, beginning on day 1 of life.

Unopened bottles of ABC Dophilus Powder from the lot received by the infant were cultured by the hospital microbiology laboratory; the samples yielded *Rhizopus* species, a mold capable of causing mucormycosis. CDC later confirmed these isolates as *Rhizopus oryzae.* Immunohistochemical staining of the cecum tissue block locally and at CDC was positive for mucormycetes. Sequencing of fungal DNA recovered from the tissue block by CDC identified the fungus as *Rhizopus oryzae*, the same species of fungus recovered from the unopened dietary supplement.

CDC, the Food and Drug Administration (FDA), and the Connecticut Departments of Health and Consumer Protection initiated an investigation. ABC Dophilus Powder, manufactured by Solgar, Inc., Leonia, New Jersey, is a dietary supplement intended to contain three live bacterial species: *Bifidobacterium lactis, Streptococcus thermophilus,* and *Lactobacillus rhamnosus* and is advertised as having probiotic effects. The dietary supplement is marketed specifically for infants and children, is available without a prescription, and is distributed widely through both wholesale and retail channels in the United States and abroad. On November 14, 2014, Solgar Inc., issued a recall (2) of several product lots, including the one fed to the infant. CDC issued public health warnings (3) advising customers and consumers not to use ABC Dophilus Powder while the investigation was ongoing.

CDC initiated intensive case-finding efforts through several large clinician, laboratory, and public health networks for infants with gastrointestinal mucormycosis or for unexplained infant deaths following receipt of Solgar ABC Dophilus. No additional cases of gastrointestinal mucormycosis in neonates have been identified to date. Case-finding also was conducted by hospital staff members in the neonatal intensive care unit that had cared for the infant; no additional infections were identified.

Dietary supplements thought to have probiotic effects have been reported to reduce the incidence of necrotizing enterocolitis and all-cause mortality in preterm infants (4), although there are concerns that the safety of the products has not been adequately documented (5). The benefits of these supplements also are being studied with a variety of other medical conditions. However, dietary supplements such as ABC Dophilus Powder are not regulated as drugs by the FDA. Therefore, these products are not subject to FDA’s premarket review and approval requirements for safety and effectiveness, nor to the rigorous manufacturing and testing standards for drugs. Rather, dietary supplements are regulated by FDA as foods with good manufacturing practice requirements specific to the commodity. Following the investigation, on December 9, 2014, FDA issued a letter encouraging health care providers who use dietary supplements containing live bacteria or yeast as drugs (including to treat or prevent medical conditions) to submit an Investigational New Drug Application to FDA for review (6). Because these products continue to be used in vulnerable populations, such as preterm infants, care must be taken to ensure the delivery of safe products to health care consumers.
